# Development of a monoclonal antibody-based competitive ELISA for serological detection of lumpy skin disease virus

**DOI:** 10.3389/fvets.2026.1819650

**Published:** 2026-05-20

**Authors:** Shaohan Li, Qiwei Zhang, Xinyin Lu, Yujie Shi, Jin Cui, Minmin Zhang, Xin Yin

**Affiliations:** 1 State Key Laboratory of Animal Disease Control and Prevention, Harbin Veterinary Research Institute, Chinese Academy of Agricultural Sciences, Harbin, China; 2 Department of Preventive Veterinary Medicine, College of Veterinary Medicine, Northeast Agricultural University, Harbin, Heilongjiang, China

**Keywords:** competitive ELISA, LSDV001, lumpy skin disease, lumpy skin disease virus, monoclonal antibody, serological surveillance

## Abstract

Lumpy skin disease (LSD) is a transboundary viral disease of cattle caused by lumpy skin disease virus (LSDV), resulting in substantial economic losses and posing a serious threat to the global cattle industry and international trade. Effective serological surveillance is essential for disease control and for evaluating vaccination programs. However, most currently available serological assays rely on a limited number of viral structural proteins as antigens, which restricts antigen diversity and may limit their applicability, particularly in the development of DIVA-compatible diagnostic strategies, highlighting the need to explore alternative diagnostic targets. In this study, we developed a monoclonal antibody-based competitive enzyme-linked immunosorbent assay (cELISA) targeting LSDV001, a highly conserved protein among Capripoxvirus species. Although LSDV001 is not a classical structural protein, it was consistently detected in purified virions and specifically recognized by antibodies in bovine immune sera, supporting its relevance as a serological target. A high-affinity monoclonal antibody (8E5) was generated and used to establish the cELISA. Receiver-operating characteristic (ROC) curve analysis showed that the sensitivity and specificity of the assay were 96.83 and 98.00%. The assay showed good reproducibility, no detectable cross-reactivity with other bovine pathogens, and strong agreement with the virus neutralization test (VNT). Further evaluation using 148 field serum samples demonstrated an overall concordance rate of 95.3% with VNT, with a Cohen's kappa coefficient of 0.913, indicating substantial agreement between the two methods. Collectively, this LSDV001-based cELISA provides a sensitive and practical tool for large-scale serological surveillance and evaluation of vaccination responses against LSD.

## 1 Introduction

Lumpy skin disease virus (LSDV), a member of the genus *Capripoxvirus* within the family *Poxviridae*, is the etiological agent of lumpy skin disease (LSD) (1–3), a highly contagious viral disease affecting cattle worldwide (4, 5). LSD is characterized by fever, skin nodules, lymphadenopathy, and a marked reduction in productivity, resulting in substantial economic losses to the livestock industry (6–9, 16–18). Owing to its rapid transboundary spread and significant impact on animal health and trade, LSD has been designated as a notifiable disease by the World Organization for Animal Health (WOAH) (10, 11). Since its first description in Zambia in 1929, LSD has progressively expanded from Africa to the Middle East, Europe, and Asia, largely driven by animal movement and cross-border trade (12–14). Therefore, robust surveillance systems and reliable diagnostic methods are essential for the effective control of outbreaks control and the implementation of vaccination strategies (15–18).

Currently, the virus neutralization test (VNT) is regarded as the gold standard for detecting antibodies against LSDV owing to its high specificity (19). However, its application is limited by its labor-intensive and time-consuming nature, as well as the requirement for biosafety level 3 (BSL-3) facilities to handle live virus, which restricts its application to specialized settings. These constraints limit its applicability in large-scale epidemiological surveillance. Although alternative serological assays, such as indirect enzyme-linked immunosorbent assays (iELISAs), have been developed, most rely on a limited number of viral structural proteins as coating antigens, including P32 (ORF074) and LSDV060 (the ortholog of vaccinia virus L1R). The P32 protein, in particular, has been widely used as a major immunodominant antigen in ELISA-based detection of LSDV antibodies. In addition, a monoclonal antibody-based cELISA targeting LSDV122 (the ortholog of vaccinia virus A33), has been reported and shown good diagnostic performance (20–22). However, these antigens are primarily encoded by essential structural genes that play critical roles in viral replication and assembly, making them difficult to manipulate for gene deletion, and thereby limiting their applicability in the development of DIVA-compatible serological assays. These limitations highlight the need to explore alternative antigen candidates that may complement existing systems and enhance serological detection strategies (23).

LSDV possesses a double-stranded linear DNA genome of approximately 151 kilobase pairs (kbp), encoding 156 predicted open reading frames (ORFs). The genome is structurally partitioned into a conserved central region and more variable terminal domains. The central region encodes the core machinery required for viral replication and morphogenesis, whereas the terminal regions are enriched in genes associated with host range determination, virulence, and immune evasion. Among these, the LSDV001/156 gene is located within the inverted terminal repeat (ITR) regions and is present in two copies (24, 25). Sequence analysis indicates that LSDV001 shares approximately 33.3% amino acid identity with homologs in vaccinia virus (VACV) and monkeypox virus (MPXV), while exhibiting high conservation (97.5%) among Capripoxvirus members (26). The LSDV001 protein is expressed during the late stage of infection and is incorparated into mature virion (MV) particles (27, 28). Compared with classical structural antigens such as P32, which are widely used due to their strong immunogenicity, LSDV001 represents a conserved virion-associated protein that is not essential for viral replication and egress based on current functional studies (29, 30), As an immunogenic protein, it has been less explored as a diagnostic antigen. Unlike classical structural proteins, such non-essential targets may be more amenable to rational modification in vaccine design, thereby offering potential advantages for DIVA-compatible serological assays. Evaluating these non-classical antigens may therefore expand the antigenic repertoire of ELISA systems and provide complementary value for serological detection.

In this study, we developed a novel monoclonal antibody (mAb)-based cELISA targeting the LSDV001 protein for the specific detection of LSDV antibodies in bovine serum (31). The cELISA format reduces non-specific binding (32) and improves diagnostic specificity (33). We describe the generation of high-affinity mAbs against LSDV001, optimization of assay conditions, and validation using both experimental and field serum samples. The developed assay demonstrated high sensitivity, specificity, and reproducibility, supporting its application in LSDV sero-surveillance and the evaluation of vaccine-induced immunity (34).

## 2 Materials and methods

### 2.1 Facility and ethics statement

All experiments involving LSDV were conducted in certified BSL-3 facilities at the Harbin Veterinary Research Institute (HVRI), Chinese Academy of Agricultural Sciences (CAAS), with approval from the Ministry of Agriculture and Rural Affairs of China. All animal-related procedures were reviewed and approved by the Institutional Animal Care and Use Committee (IACUC) of HVRI (Approval No. 250926-02-GR) and were performed in strict accordance with the guidelines outlined in the *Guide for the Care and Use of Laboratory Animals* issued by the Ministry of Science and Technology of the People's Republic of China.

### 2.2 Cells and cell culture

Lamb testicular (LT) cells were maintained in Eagle's Minimum Essential Medium (MEM; Gibco, USA) supplemented with 10% fetal bovine serum (FBS; Gibco, Thermo Fisher Scientific, USA) and 1% penicillin–streptomycin. Human embryonic kidney 293T cells expressing the SV40 large T antigen (HEK293T) and African green monkey kidney (Vero E6) cells were cultured in Dulbecco's Modified Eagle's Medium (DMEM; Gibco, USA) supplemented with 10% FBS and 1% penicillin-streptomycin. *Escherichia coli* DH5α cells (TIANGEN, China) were used for plasmid construction, amplification, and transformation. *E. coli* Rosetta (DE3) cells (TIANGEN, China) were used for recombinant protein expression.

### 2.3 Plasmids, virus, and serum samples

The pCold-6× His expression vector, pCAGGS-Flag vector, and the recombinant LSDV-TK-GFP virus were maintained in our laboratory. Serum samples seropositive for LSDV, bovine coronavirus (BCoV), infectious bovine rhinotracheitis virus (IBRV), bovine rotavirus (BRV), Akabane virus (AKAV), and bovine leukemia virus (BLV) were collected from cattle with confirmed serological status following vaccination or field exposure.

### 2.4 Reagents and antibodies

The reagents used in this study included isopropyl β-D-1-thiogalactopyranoside (IPTG; I8070, Solarbio, China), high-affinity Ni–NTA resin (L00250, GenScript, China), lysis–elution (LE) buffer (50 mm NaH_2_PO_4_, 300 mm NaCl, pH 8.0), Quick Blue Rapid Protein Stain (BF06152, Biodragon, China), polyethylene glycol/dimethyl sulfoxide solution (P7306, Sigma, USA), hypoxanthine–aminopterin–thymidine (HAT) medium supplement (50×; H0262, Sigma, USA), Protein G resin (L00209, GenScript, China), and a monoclonal antibody isotype identification enzyme ready-to-use kit (BF16002X, Biodragon, China).

A 6× His tag monoclonal antibody (1:25,000; 66005-1-Ig, Proteintech, China) was used in this study. Secondary antibodies included horseradish peroxidase (HRP)-conjugated goat anti-mouse IgG (Fc-specific; 1:5,000; A2554, Sigma, USA), Alexa Fluor 488-conjugated goat anti-mouse IgG (H + L; 1:1,000; A28175, Invitrogen, USA), and Alexa Fluor 568-conjugated goat anti-mouse IgG (H + L; 1:1,000; A11004, Invitrogen, USA).

### 2.5 Recombinant LSDV001 expression and purification

The full-length ORF of the LSDV001 gene was amplified from the LSDV isolate China/Xinjiang/Cattle/Aug-2019 strain (GenBank: OP508345) and cloned into the pCold-6× His expression vector using the *BamHI* and *HindIII* restriction sites. The resulting recombinant plasmid was transformed into *E. coli* Rosetta (DE3) cells. Recombinant protein expression was induced with 0.2 mm IPTG for 16 h at 16 °C.

Following induction, bacterial cells were harvested by centrifugation, resuspended in LE buffer, and lysed by sonication. The lysate was clarified by centrifugation at 12,000 × g for 15 min at 4 °C, and the resulting supernatant containing the LSDV001–6 × His fusion protein was loaded onto a Ni–NTA affinity chromatography column. Non-specifically bound proteins were removed by washing with LE buffer supplemented with 40 mm imidazole, and the target protein was eluted using LE buffer containing 250 mm imidazole.

The purified protein was dialyzed overnight at 4 °C against 0.02 M phosphate-buffered saline (PBS) to remove residual imidazole. Successful expression and purification of LSDV001 were confirmed by sodium dodecyl sulfate-polyacrylamide gel electrophoresis (SDS–PAGE) and western blot analysis using a commercial anti-His tag monoclonal antibody and LSDV-positive antiserum.

### 2.6 Production of LSDV001 monoclonal antibodies

All animal experiments in this study were approved from the Animal Ethics Committee of Harbin Veterinary Research Institute of the Chinese Academy of Agricultural Sciences. All procedures were conducted in accordance with the local legislation and institutional requirements. Mice were immunized three times with the antigen at days 0, 21, and 42. 1 week after the final immunization, the mice were euthanized and splenocytes were harvested. The isolated splenocytes were fused with SP2/0 myeloma cells using polyethylene glycol, following standard hybridoma production procedures.

The fused cells were cultured in RPMI 1640 medium supplemented with 20% FBS and 1 × HAT solution. Cell suspensions were distributed into 96-well plates and incubated at 37 °C in a humidified atmosphere containing 5% CO_2_. Supernatants from the resulting hybridoma cultures were screened by iELISA using LSDV001 as the coating antigen. Hybridomas exhibiting positive reactivity were subjected to three rounds of limiting dilution cloning to ensure monoclonality.

For monoclonal antibody production, 2 × 10^6^ cloned hybridoma cells were injected intraperitoneally into BALB/c mice that had been pretreated with liquid paraffin 1 week prior to inoculation. mAbs were produced *in vivo* and purified by Protein G affinity chromatography. The purified mAbs were analyzed by SDS–PAGE, and their immunoglobulin subclasses and light chain types were determined using a commercial monoclonal antibody isotyping kit.

### 2.7 Indirect immunofluorescence assay

HEK293T and Vero E6 cells were seeded in 96-well plates at a density of 1 × 10^4^ cells per well. HEK293T cells were transfected with the pCAGGS-LSDV001 plasmid, whereas Vero E6 cells were infected with LSDV-TK-GFP at a multiplicity of infection (MOI) of 0.1.

Following transfection or infection, the cells were fixed with 4% paraformaldehyde for 15 min at room temperature and permeabilized with 0.2% Triton X-100. After blocking with 1% bovine serum albumin (BSA; Solarbio, China), the cells were incubated with LSDV001-specific mAbs for 1 h at 37 °C. The cells were then washed with PBS and incubated with Alexa Fluor 488- or 568-conjugated goat anti-mouse IgG secondary antibodies. After three additional washes with PBS, fluorescence signals were visualized using an EVOS imaging system (Life Technologies, USA).

### 2.8 Transient transfection and LSDV001 protein expression in HEK293T cells

The pCAGGS-LSDV001 expression plasmid was constructed by inserting the full-length ORF001 into the pCAGGS vector between the *EcoRI* and *NheI* restriction sites. A 6 × His tag and a Flag tag were fused to the C-terminus of LSDV001 to facilitate detection. The recombinant plasmid was transiently transfected into HEK293T cells for protein expression analysis. Briefly, cells were seeded into 6-well plates and cultured to approximately 70%−80% confluence at the time of transfection. The pCAGGS-LSDV001 plasmid was transfected using polyethylenimine (PEI) transfection reagent (Polysciences, Warrington, PA, USA) according to the manufacturer's instructions. For each well, the appropriate amount of plasmid DNA was diluted in serum-free medium, mixed with transfection reagent, and incubated for 15 min at room temperature to allow complex formation before being added dropwise to the cells.

Following transfection, cells were incubated for 48 h and then lysed using NP-40 lysis buffer supplemented with protease inhibitors (Beyotime Biotechnology, China), followed by centrifugation to remove cellular debris. The clarified supernatants containing expressed proteins were subsequently evaluated by SDS–PAGE and western blotting using monoclonal antibodies as primary antibodies, as described below.

### 2.9 Indirect ELISA (iELISA)

Purified LSDV001 protein was diluted in carbonate–bicarbonate buffer (CBS) to a final concentration of 2.5 μg/ml and coated onto 96-well plates (100 μl per well; Corning, USA) overnight at 4 °C. The plates were washed three times with PBST (0.01 M PBS containing 0.05% Tween-20) and blocked with 5% skimmed milk (BD, USA) in PBST for 1 h at 37 °C.

After washing, 100 μl of hybridoma culture supernatant was added to each well and incubated for 1 h at 37 °C. Sera from LSDV001-immunized and non-immunized mice, diluted 1:1,000, were used as positive and negative controls, respectively. Following three washes, 100 μl of HRP-conjugated goat anti-mouse IgG was added and incubated for 1 h at 37 °C.

After additional washing, 100 μl of tetramethylbenzidine (TMB) substrate (Biodragon, China) was added to each well, and the reaction was allowed to proceed for 10 min at room temperature before being stopped with 100 μl of 2 M H_2_SO_4_. The optical density at 450 nm (OD_450_) was measured using a microplate reader (BioTek, USA).

### 2.10 Virus neutralization test

Serum samples were tested for LSDV-neutralizing antibodies according to the *WOAH Manual* of Diagnostic Tests and Vaccines for Terrestrial Animals. All sera were heat-inactivated at 56 °C for 30 min and then serially diluted two-fold starting from 1:4. Each serum dilution was mixed with 100 TCID_50_ of LSDV per well and incubated at 37 °C for 1 h in 96-well microplates (100 μl per well).

Subsequently, 1.5 × 10^4^ LT cells were added to each well, and the plates were incubated at 37 °C for 5 days. Each assay included positive and negative serum controls, as well as virus-infected and uninfected cell controls. Cytopathic effects (CPE) were examined at 5 days post-infection (dpi) using an inverted microscope.

Neutralizing antibody titers were calculated using the Reed–Muench method and defined as the highest serum dilution resulting in 50% inhibition of virus-induced CPE. Serum samples with neutralizing activity detected at a dilution of ≥1:4 were considered positive whereas samples below this threshold were regarded as negative.

### 2.11 Serum panel

Between April 2024 and December 2025, a total of 113 bovine serum samples were collected from four cattle farms located in Heilongjiang Province and the Xinjiang Uygur Autonomous Region. Positive reference sera (*n* = 50) were obtained from cattle vaccinated with a commercial goatpox live vaccine and were confirmed as LSDV antibody-positive by VNT. Negative sera (*n* = 63) were collected from LSDV-free farms with no history of LSDV vaccination or clinical signs. These well-characterized VNT-positive and VNT-negative sera were used to establish the diagnostic cut-off value for the LSDV001-based cELISA.

To evaluate the performance of the LSDV001 cELISA in detecting vaccine-induced immune responses, an additional 148 bovine serum samples were collected in October 2025 from three cattle farms in Heilongjiang Province, including 70 samples from vaccinated cattle and 78 samples from VNT-confirmed seropositive animals. These field-collected samples were also obtained from cattle vaccinated with a commercial goatpox live vaccine. Prior to analysis, all serum samples were heat-inactivated at 56 °C for 30 min. No live virus was handled during serum processing.

### 2.12 Development and optimization of the cELISA

To evaluate the competitive capacity of the mAbs, a cELISA was initially performed using five LSDV-positive and five LSDV-negative serum samples. Briefly, 96-well microplates were coated overnight at 4 °C with purified LSDV001 antigen (100 μl per well) diluted to 2.5 μg/ml in CBS. The plates were washed three times with PBST and blocked with 200 μl per well of 5% skimmed milk in PBST for 1 h at 37 °C.

Subsequently, 50 μl of each test serum sample was mixed with 50 μl of monoclonal antibody solution and added to the corresponding wells, followed by incubation for 1 h at 37 °C. After washing three times with PBST, 100 μl of HRP-conjugated goat anti-mouse IgG (1:5,000 diluted in PBST) was added to each well and incubated for 1 h at 37 °C. Following a final washing step, 100 μl of TMB substrate was added for color development, and the reaction was stopped with 100 μl of 2 M H_2_SO_4_. The optical density was measured at 450 nm (OD_450_) using a microplate reader.

The percent inhibition (PI) was calculated using the following formula:


$$\begin{array}{l} PI\left(\%\right)=\;\left[\frac{\left(mean\;OD\;of\;negative\;control\;-\;OD\;of\;sample\right)}{mean\;OD\;of\;negative\;control}\;\right]\times \;100 \end{array}$$

For assay optimization, various concentrations of LSDV001 antigen (0.0625–10 μg/ml) and serial dilutions of mAb 8E5 (1:200–1:25,600) were evaluated. Optimal conditions were determined by targeting an OD_450_ value of approximately 1.0 for the positive control in the iELISA. Subsequently, LSDV antibody-positive and -negative serum samples were tested at dilutions ranging from 1:1 to 1:64, and the optimal serum dilution was selected based on the lowest positive-to-negative (P/N) ratio. Incubation times for sera and mAb 8E5 were assessed at 30, 60, 90, and 120 min, while TMB color development times were evaluated at 5, 10, 15, and 20 min. The final assay conditions were selected to minimize the P/N ratio and maximize discrimination between positive and negative reference sera.

### 2.13 Validation of the LSDV001 cELISA

To evaluate the specificity of the LSDV001 cELISA, bovine serum samples were collected from cattle that had been previously infected with or vaccinated against different bovine viruses, including LSDV, BVDV, BCoV, IBRV, BRV, AKAV, and BLV.

The reproducibility of the cELISA was assessed using a subset of eight serum samples, consisting of four LSDV-positive and four LSDV-negative sera. Analytical consistency was evaluated by calculating the coefficient of variation (CV), expressed as CV (%) = (standard deviation / mean) × 100. Intra-assay precision was determined by testing each serum sample in triplicate within the same plate, whereas inter-assay precision was evaluated by testing the same samples in three independent assay runs performed on different days.

### 2.14 Immunization of calves and serum sample collection

All virus propagation and inactivation procedures were conducted under BSL-3 conditions. LT cells were seeded in T225 flasks at a density of 5 × 10^7^ cells per flask and infected with LSDV at a MOI of 0.1. At 5 days post-infection (dpi), viruses were harvested from both cell lysates and culture supernatants by three consecutive freeze–thaw cycles. The lysates were clarified by centrifugation at 250 × g for 15 min to remove cellular debris, and the viral titer was determined using the 50% tissue culture infectious dose (TCID_50_) assay.

For virus inactivation, LSDV was treated with binary ethyleneimine (BEI) at a final concentration of 4 mm for 24 h at 30 °C. The inactivated virus was subsequently emulsified with the oil-based adjuvant ISA 15A VG at a volume ratio of 85:15 (v/v). Eight LSDV-seronegative calves aged 3–6 months were randomly assigned to either an immunization group (*n* = 5) or a control group (*n* = 3). Calves in the immunization group received a primary intramuscular injection in the neck region with 1 ml of inactivated LSDV vaccine containing 10^7^ TCID_50_/ml. Booster immunizations were administered at 2–4 weeks after the primary vaccination. Blood samples were collected from all calves at 2-week intervals following the initial immunization.

### 2.15 Statistical analysis

Receiver operating characteristic (ROC) curve analysis was performed using a total of 50 LSDV antibody-positive and 63 LSDV antibody-negative serum samples to determine the diagnostic cut-off value, as well as the sensitivity and specificity of the LSDV001 cELISA. Statistical analyses and graphical representations were performed using GraphPad Prism software (version 9.4.1; GraphPad Software, Inc., La Jolla, CA, USA).

Agreement between the cELISA and the VNT was assessed by calculating Cohen's kappa coefficient using Microsoft Excel. The kappa coefficient was interpreted as follows: values >0.75 indicate excellent agreement, 0.40–0.75 indicate moderate agreement, and < 0.40 indicate poor agreement.

## 3 Results

### 3.1 Characterization and purification of the LSDV001 protein

**Figure 1:**
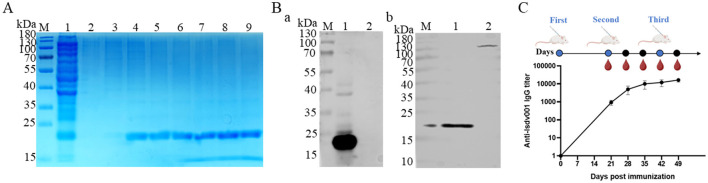
Expression of LSDV001 protein and assessment of immunogenicity. **(A)** SDS–PAGE analysis of LSDV001, M, protein marker; lane 1, bacterial lysate supernatants induced by 0.2 mm IPTG; lanes 2–9, purified LSDV001 protein. **(B)** Western blotting analysis of purified LSDV001 protein probed with different antibodies. (a) Incubated with anti-His tag mouse monoclonal antibody; (b) Incubated with LSDV positive bovine serum. M, protein marker; lane 1, purified LSDV001 protein; lane 2, blank bacterial cell lysate. **(C)** Immunization schedule of BALB/c mice and iELISA analysis of anti-LSDV001 sera.

The LSDV001 gene was cloned into the pCold-6 × His expression vector and transformed into *E. coli* Rosetta (DE3) cells. Recombinant LSDV001 was successfully expressed in a soluble form and purified using Ni^2+^-NTA affinity chromatography. SDS–PAGE analysis revealed a predominant protein band at approximately 18.8 kDa, consistent with the predicted molecular weight of LSDV001 (Figure 1A). Western blot analysis further confirmed the antigenic specificity of the purified protein, as a single band at the expected molecular weight was recognized by both an anti-His tag mouse monoclonal antibody and bovine LSDV-positive serum (Figure 1B).

To evaluate the immunogenicity of LSDV001, BALB/c mice were immunized with the recombinant protein, and serum samples were collected on days 0, 21, 35, 42, and 49 post-immunization. As shown in Figure 1C, anti-LSDV001 IgG titers increased progressively and reached a peak endpoint titer of approximately 1:100,000. These results indicate that LSDV001 is immunogenic, supporting its suitability as a candidate antigen for serological diagnostic development.

### 3.2 Generation and characterization of monoclonal antibodies against LSDV001

**Figure 2:**
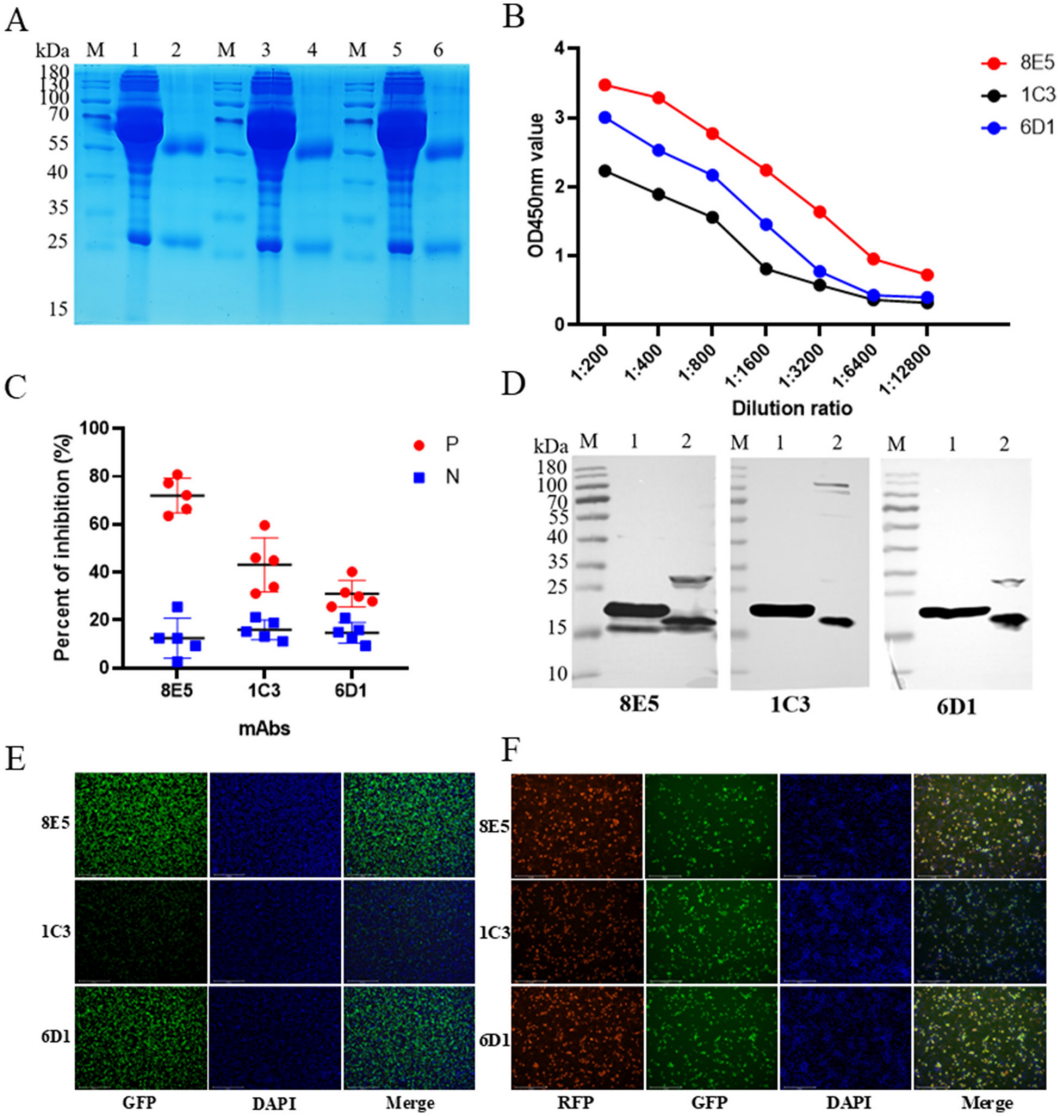
Characterization of anti-LSDV001 monoclonal antibodies 8E5, 1C3, and 6D1. **(A)** SDS–PAGE analysis of monoclonal antibodies before and after purification. M, protein marker; lane 1, mAb 8E5 pre-purification; lane 2, mAb 8E5 post-purification; lane 3, mAb 1C3 pre-purification; lane 4, mAb 1C3 post-purification; lane 5, mAb 6D1 pre-purification; lane 6, mAb 6D1 post-purification. **(B)** Reactivity of monoclonal antibodies with LSDV001 determined by iELISA. **(C)** Ability of monoclonal antibodies to differentiate LSDV-positive and negative sera evaluated by cELISA. Five LSDV-positive sera (red) and five negative sera (blue) were tested, and the percentage inhibition (PI) values were calculated. **(D)** Western blot analysis of prokaryotically and eukaryotically expressed LSDV001 proteins probed with monoclonal antibodies 8E5, 1C3, and 6D1. M, protein marker; lane 1, prokaryotically expressed LSDV001; lane 2, eukaryotically expressed LSDV001. **(E)** Indirect immunofluorescence assay (IFA) showing reactivity of monoclonal antibodies with eukaryotically expressed LSDV001 protein in transfected HEK293T cells. GFP fluorescence indicates recombinant LSDV001 expression, and nuclei were counterstained with DAPI. **(F)** Indirect immunofluorescence assay of Vero-E6 cells infected with recombinant LSDV-TK-GFP. Red fluorescence represents binding of monoclonal antibodies, green fluorescence indicates GFP expression from recombinant virus, and nuclei were stained with DAPI. Co-localization of red and green signals confirms specific recognition of LSDV-infected cells by the monoclonal antibodies.

mAbs against LSDV001 were generated using standard hybridoma technology as previously described (35). Three hybridoma clones exhibiting specific reactivity to LSDV001 were obtained and designated 8E5, 1C3, and 6D1. Isotype analysis revealed that all three mAbs belonged to the IgG1 subclass with κ light chains. The mAbs were purified from mouse ascitic fluid, and SDS–PAGE analysis confirmed high purity, showing heavy chains at approximately 53 kDa and light chains at approximately 25 kDa (Figure 2A).

The binding activity of the mAbs was evaluated by iELISA. Two-fold serial dilutions were prepared from an initial concentration of 2.5 μg/ml, starting at 1:200. Among the three mAbs, 8E5 exhibited the highest antibody titer and the strongest reactivity against the LSDV001 antigen (Figure 2B). The competitive binding capacity of the mAbs was further assessed using a cELISA with five LSDV-positive and five LSDV-negative bovine serum samples. The results showed that all five positive sera inhibited the binding of mAb 8E5 by more than 63.53%, with a mean PI value of 72.06%, whereas the mean PI value for negative sera was only 12.45%. In contrast, the mean PI value for mAbs 1C3 and 6D1 with positive sera were 43.13 and 31.02%, respectively (Figure 2C). These findings indicate that mAb 8E5 possessed the strongest competitive binding capability among the three mAbs.

Western blot analysis further demonstrated that mAb 8E5 specifically recognized the LSDV001 protein expressed in both prokaryotic and eukaryotic systems. As shown in Figure 2D, mAb 8E5 detected a clear band corresponding to the expected molecular weight of LSDV001 (approximately 18.8 kDa) in lysates derived from *E. coli* and transfected eukaryotic cells, whereas no non-specific bands were observed, indicating high specificity and strong reactivity.

To further evaluate the reactivity of mAb 8E5 toward LSDV001 expressed in eukaryotic cells, HEK293T cells were transfected with the pCAGGS-LSDV001 expression plasmid, and mAb 8E5 produced specific fluorescence signals that co-localized with the LSDV001 protein, whereas no specific signal was detected in control cells (Figure 2E).

In addition, IFA conducted on Vero E6 cells infected with recombinant LSDV-TK-GFP further confirmed the specificity of mAb 8E5 for native viral antigen. As shown in Figure 2F, robust red fluorescence corresponding to mAb 8E5 binding was observed and showed clear co-localization with GFP-expressing LSDV-infected cells, indicating that mAb 8E5 recognizes LSDV001 in the context of viral infection.

Based on its high reactivity and superior competitive inhibition capacity, mAb 8E5 was selected as the optimal detection antibody for subsequent development of the cELISA.

### 3.3 Establishment and optimization of the LSDV001-based cELISA

**Figure 3:**
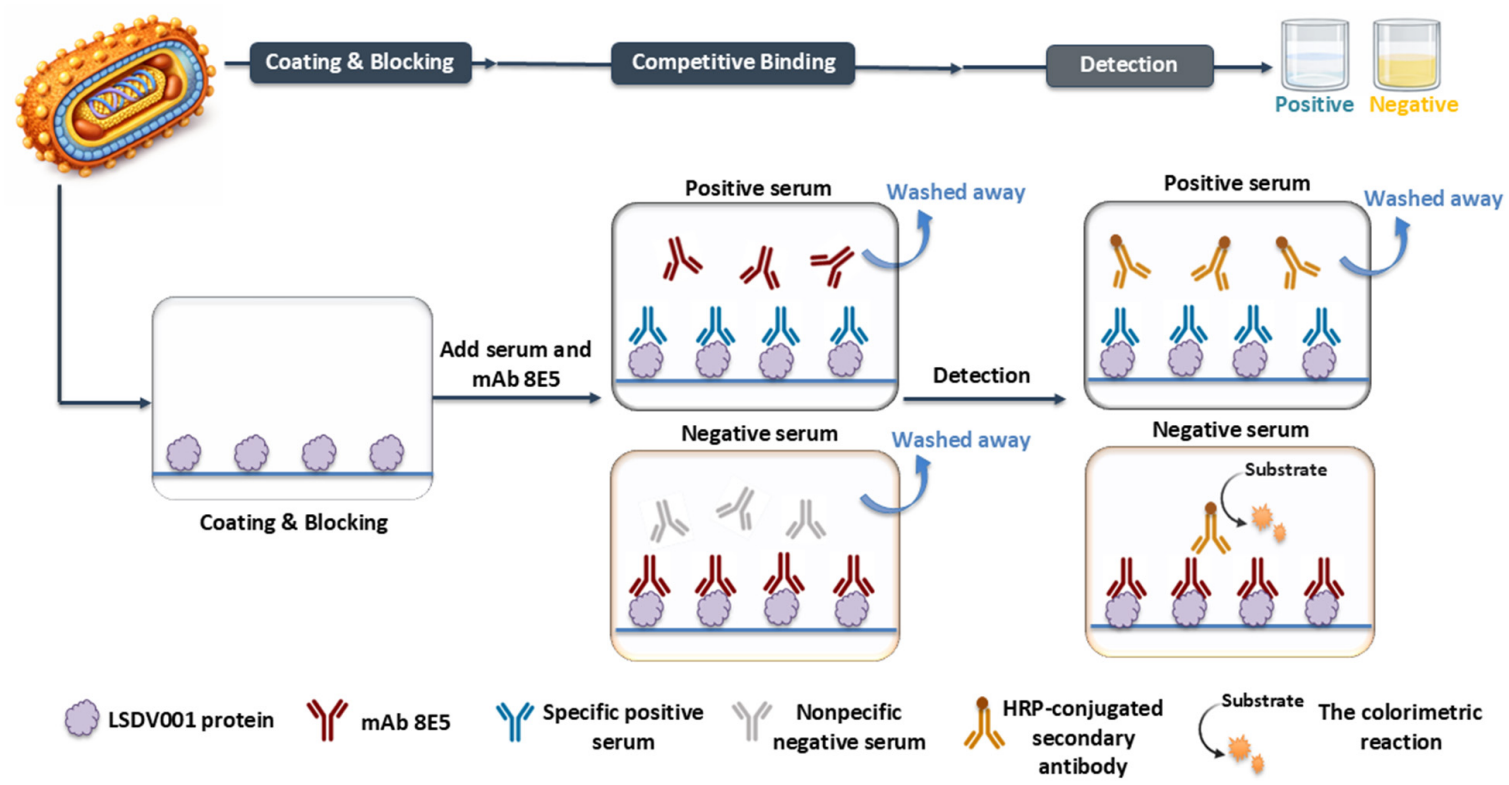
Schematic diagram illustrating the design and workflow of the LSDV001-based monoclonal antibody competitive ELISA (cELISA) developed in this study.

Checkerboard titration was performed to optimize the assay conditions. The optimal working dilution of mAb 8E5 was determined to be 1:6,400 (corresponding to 0.5 μg/ml), and the optimal coating concentration of the LSDV001 antigen was 1.25 μg/ml. Under these conditions, the optimal serum dilution was identified as 1:2. Further optimization showed that the optimal incubation times were 1 h for both the serum samples and mAb 8E5, and 10 min for the colorimetric reaction. These conditions were defined as the optimized parameters for the LSDV001 cELISA (Table 1), which was subsequently used for diagnostic evaluation (Figure 3). Detailed optimization results, including checkerboard titration and condition optimization, are provided in the Supplementary Materials.

**Table 1:** Optimization of assay conditions for the LSDV001-based cELISA.

Optimized dilutions and reaction conditions	LSDV001 cELISA
Coating condition	1.25 μg/ml in CBS
	4 °C, 12 h
Blocking condition	5% skimmed milk
	37 °C, 1 h
Serum samples and mAb 8E5	1:2 and 1:6400 (0.5 μg/ml), respectively
HRP-conjugated secondary antibody	1:5000
	37 °C, 1 h
Chromogenic substrate	100 μl/well
	RT, 10 min

**Figure 4:**
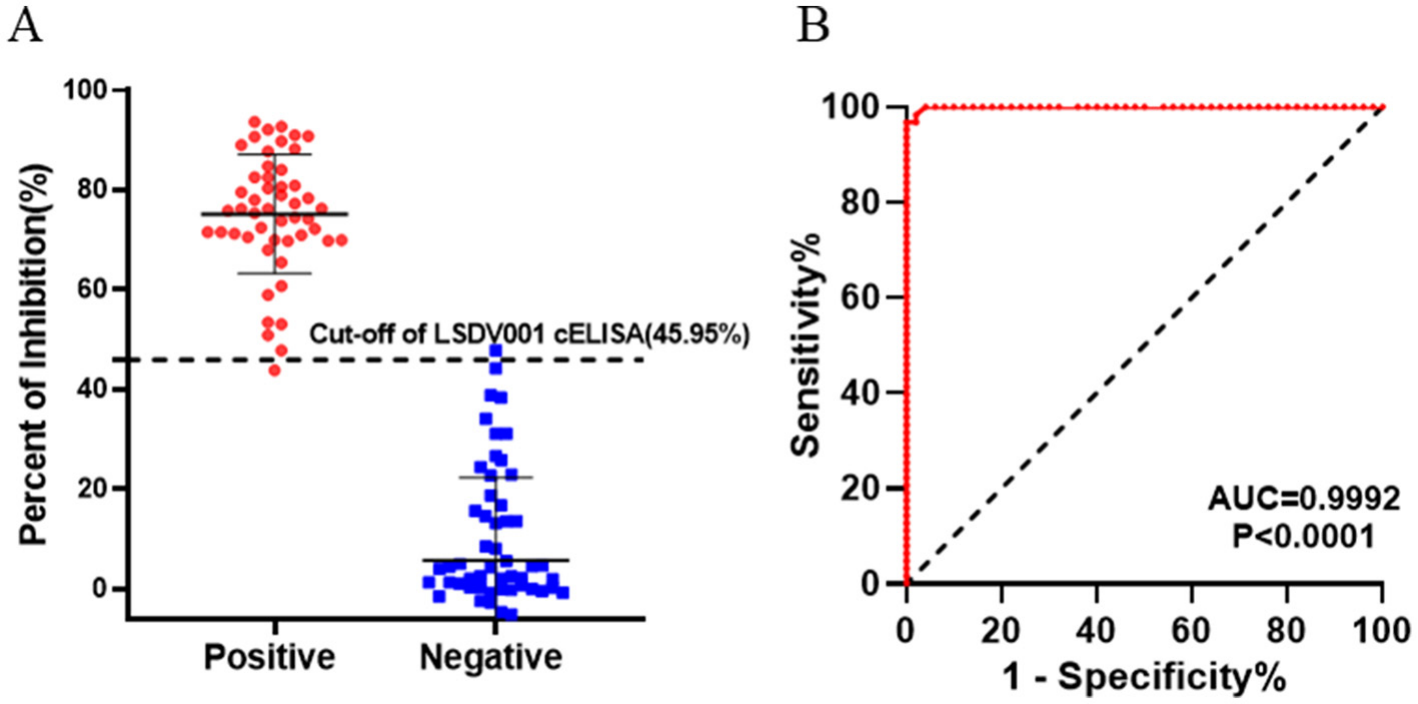
Receiver operating characteristic (ROC) analysis for the LSDV001 cELISA. The assay was conducted using LSDV-negative sera (*n* = 63) and LSDV-positive sera (*n* = 50). **(A)** Scatter plot displaying PI value of individual serum samples, with the cut-off value set at 45.95 %. **(B)** ROC curve analysis of cELISA, yielding an area under the curve (AUC) of 0.9992.

To determine the diagnostic cut-off value as well as the sensitivity and specificity of the assay, the cELISA was evaluated using a panel of 63 LSDV-negative and 50 LSDV-positive serum samples. The PI value for each sample were calculated and presented in a scatter plot (Figure 4A). ROC curve analysis was then conducted to identify the optimal PI cut-off value that maximized diagnostic sensitivity and specificity (Figure 4B). The ROC analysis yielded an area under the curve (AUC) of 0.9992 (95% confidence interval [CI]: 0.9973–1.000). When a PI cut-off value of 45.95% was applied, the assay achieved a diagnostic sensitivity of 96.83% (95% CI: 0.8914–0.9944) and a diagnostic specificity of 98.00% (95% CI: 0.8950–0.9999). Accordingly, serum samples with PI value < 45.95% were classified as negative, whereas those with PI value ≥ 45.95% were classified as positive. Notably, one false-positive sample (*PI* = 47.83%) was observed among the 63 negative sera, and one false-negative sample (*PI* = 43.82%) was detected among the 50 positive sera.

### 3.4 Specificity and sensitivity of the LSDV001 cELISA

**Figure 5:**
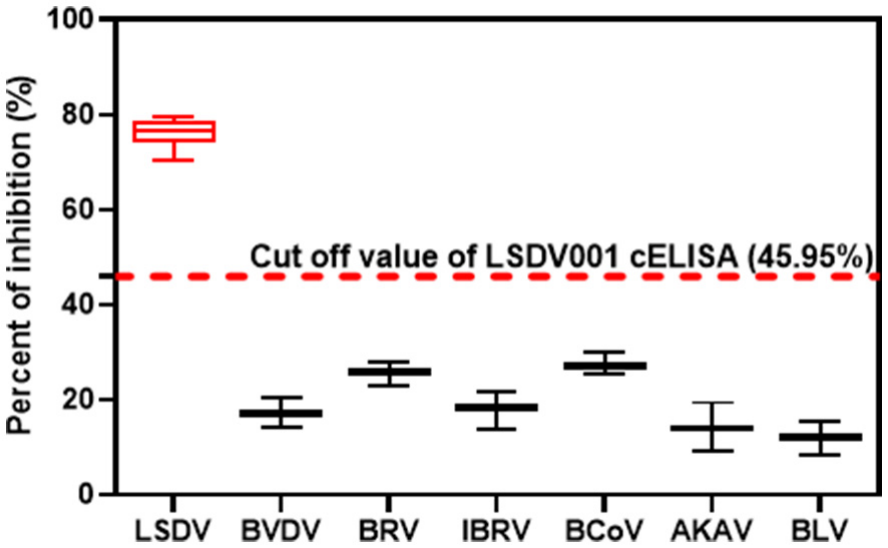
Specificity of the LSDV001-based cELISA. Specificity of the cELISA was evaluated using sera positive for LSDV and other bovine viruses, including bovine viral diarrhea virus (BVDV), bovine rotavirus (BRV), infectious bovine rhinotracheitis virus (IBRV), bovine coronavirus (BCoV), Akabane virus (AKAV), and bovine leukemia virus (BLV). The horizontal dashed line indicates the cut-off value.

The analytical specificity of the LSDV001 cELISA was evaluated using sera positive for LSDV and for other common bovine pathogens, including BVDV, BRV, IBRV, BCoV, AKAV, and BLV. All sera positive for non-LSDV bovine viruses exhibited PI value below the established cut-off of 45.95%, whereas all LSDV-positive sera exceeded this threshold, indicating that the assay is highly specific for the detection of LSDV antibodies and shows no detectable cross-reactivity with antibodies against other bovine viruses (Figure 5).

**Figure 6:**
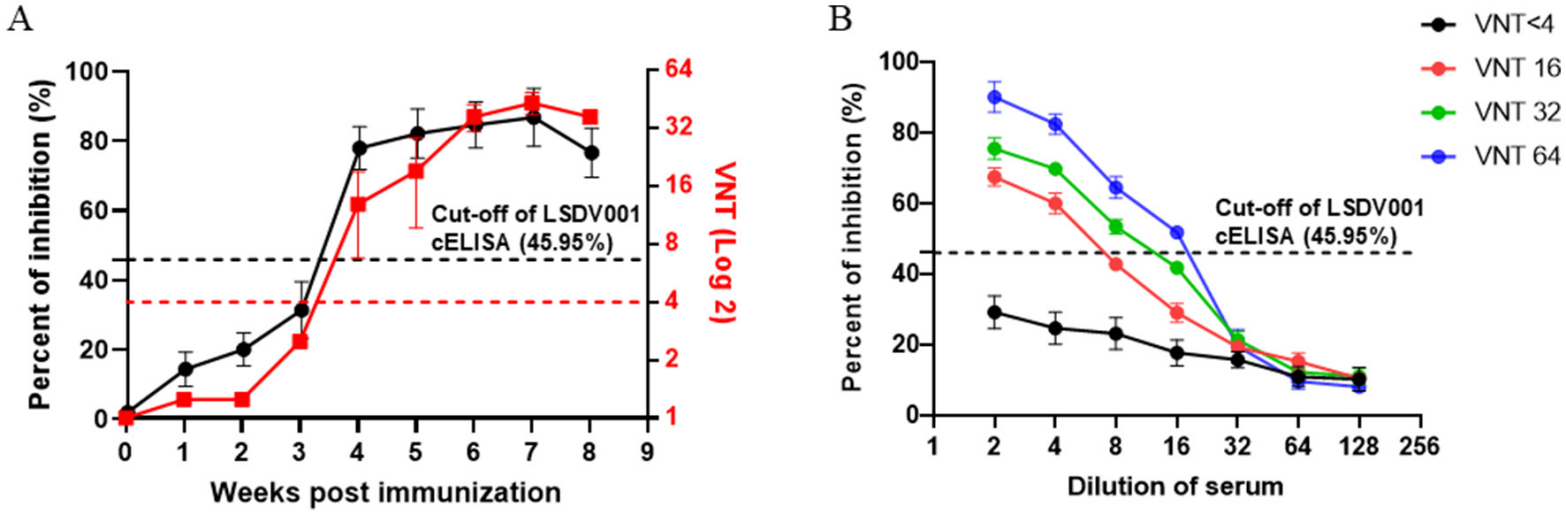
Comparison of antibody dynamics detected by the LSDV001-based cELISA and virus neutralization test (VNT) following LSDV immunization. **(A)** Kinetics of antibody responses in immunized animals as determined by the LSDV001 cELISA and the VNT. The black dashed line indicates the cut-off value and the red dashed line indicates the cut-off titer of VNT. **(B)** Detection of the serially diluted sera with different neutralizing antibody titers by the LSDV001-based cELISA. The black dashed line indicates the cut-off value.

To further assess the analytical sensitivity of the assay, three calves were immunized with inactivated LSDV, and serum samples collected at different post-vaccination time points were tested using both the LSDV001 cELISA and VNT. As shown in Figure 6A, seroconversion was first detected at 4 weeks post-vaccination by both assays, and comparable antibody kinetic profiles were observed. In addition, four sera with different neutralizing antibody titers (VNT < 4, 16, 32, and 64) were subjected to two-fold serial dilution and analyzed by cELISA. The PI value decreased progressively with increasing dilution, and the highest dilutions that remained positive in the cELISA were 1:8, 1:16, and 1:32 for sera with VNT titers of 16, 32, and 64, respectively (Figure 6B). These results indicated that the LSDV001 cELISA effectively reflects LSDV-specific antibody dynamics and that PI value are closely correlated with neutralizing antibody titers.

### 3.5 Repeatability and reproducibility of the LSDV001 cELISA

The analytical precision of the LSDV001 cELISA was evaluated using four LSDV-positive and four LSDV-negative serum samples. To assess repeatability (intra-assay variation), each sample was tested in triplicate within the same plate. Reproducibility (inter-assay variation) was evaluated by testing each sample in three independent assay runs performed on different days.

The coefficients of variation (CVs) for PI value ranged from 0.80 to 4.46% for intra-assay measurements and from 1.70 to 4.68% for inter-assay measurements (Table 2). All CV values were below 10%, indicating that the LSDV001 cELISA exhibits excellent repeatability, reproducibility, and analytical reliability.

**Table 2:** Repeatability of the LSDV001-based cELISA.

Samples	Repeatability (intra-assay)			Reproducibility (inter-assay)		
	Mean PI (%)	SD	CV (%)	Mean PI (%)	SD	CV (%)
1	90.02	1.08	1.20	86.11	3.80	4.41
2	89.71	2.44	2.72	80.27	2.92	3.63
3	85.13	0.68	0.80	82.93	2.66	3.20
4	69.94	0.96	1.38	69.18	1.18	1.70
5	25.45	1.13	4.46	27.25	0.77	2.83
6	34.34	0.61	1.77	35.32	1.05	2.96
7	11.65	0.4	3.45	13.04	0.58	4.45
8	22.50	0.50	2.20	23.12	1.08	4.68

### 3.6 Clinical performance of the LSDV001 cELISA

To evaluate the clinical diagnostic performance of the LSDV001 cELISA, a total of 148 serum samples, including 70 samples from vaccinated cattle and 78 samples from VNT-confirmed seropositive animals, were tested in parallel using the cELISA and the VNT. The results showed an overall concordance rate of 95.3% between the two methods. Statistical analysis indicated a high level of agreement between the LSDV001 cELISA and VNT, with a Cohen's kappa coefficient of 0.913 (Table 3), indicating substantial agreement between the two diagnostic methods. These results demonstrate that the LSDV001 cELISA is highly consistent with the reference VNT and confirm its suitability for clinical application and large-scale serological surveillance.

**Table 3:** Agreement between the LSDV001-based cELISA and VNT.

cELISA result	VNT result		Total	Agreement (%)	Kappa value
	Positive	Negative			
Positive	63	0	63	95.3	0.913
Negative	7	78	85		
Total	70	78	148		

## 4 Discussion

The WOAH Terrestrial Manual (2023) recommends ELISA as a preferred method for monitoring LSDV infection and immune responses (36), owing to its simplicity and suitability for large-scale application (11). In this study, we developed and validated a monoclonal antibody-based cELISA for the detection of LSDV antibodies using recombinant LSDV001 antigen and a highly specific monoclonal antibody (mAb 8E5). The assay showed high specificity, with no detectable cross-reactivity with antisera against common bovine viruses, and exhibited strong agreement with VNT in clinical sample evaluation. In addition, it effectively tracked antibody dynamics following immunization, supporting its applicability across different stages of infection or vaccination.

The selection of LSDV001 as the target antigen was critical to the performance of the assay. LSDV001 is a conserved virion-associated protein that elicited a detectable humoral immune response in immunized animals. Importantly, the recombinant LSDV001 protein expressed in *E. coli* retained antigenic epitopes recognized by both polyclonal sera and monoclonal antibodies, supporting its suitability for serological detection. The selected mAb 8E5 exhibited high affinity and strong competitive inhibition capacity, and showed no cross-reactivity with antibodies against other bovine pathogens, including BVDV, BCoV, IBRV, BRV, AKAV, and BLV. This level of specificity is particularly relevant for field applications, where co-infections or prior vaccinations may complicate interpretation of serological results.

Compared with conventional serological methods, the LSDV001-based cELISA provides several practical advantages. The assay does not require handing of live virus, thereby reducing biosafety risks and enabling implementation in routine diagnostic laboratories. In addition, the cELISA format reduces non-specific binding, allowing reliable detection of LSDV-specific antibodies in both infected and vaccinated animals (37, 38). In the present study, an indirect detection format (unlabeled primary mAb combined with an HRP-conjugated secondary antibody) was employed, enabling rapid assay establishment without additional conjugation or re-optimization steps. Although this format requires a slightly longer incubation time compared with assays using HRP-labeled primary antibodies, the total assay time (~2.5 h) remains acceptable for routine laboratory applications. The assay also demonstrated good repeatability and reproducibility, with intra- and inter-assay variation consistently below 10%.

Antibody kinetics observed in experimentally immunized calves were consistent with VNT results, supporting its utility for monitoring seroconversion and post-vaccination responses (39, 40). In this study, diagnostic sensitivity and specificity were initially determined by ROC curve analysis using a defined training set, whereas concordance with VNT was further evaluated using an independent panel of field serum samples. In the validation set, a small number of VNT-positive sera were not detected by the cELISA, resulting in a lower apparent sensitivity. This discrepancy is likely related to differences in sample sources. Sera from experimentally immunized animals generally exhibit relatively high and homogeneous antibody responses, whereas field samples may present greater variability in antibody levels, immune status, and infection history. Notably, most discrepant sera had low VNT titers (data not shown), suggesting that low antibody levels may contribute to reduced detection sensitivity. In addition, VNT detects functional neutralizing antibodies, whereas the cELISA is based on a single antigen (LSDV001). Therefore, samples with low antibody levels or with antibody profiles not strongly directed against LSDV001 may not be detected, resulting in apparent false-negative results. Future studies integrating multiple antigen targets into a combined detection system may improve sensitivity and reduce discordance, particularly when testing heterogeneous field samples. In addition, preparation of HRP-labeled mAb will be explored in future work to further simplify the assay procedure and facilitate high-throughput or field applications.

Most currently available ELISA assays for LSDV antibody detection rely on a limited number of structural proteins, particularly P32. While these assays generally show good diagnostic performance, their dependence on highly conserved structural proteins may restrict their applicability in certain contexts, including DIVA-based strategies and differentiation among closely related Capripoxvirus infections. Moreover, these structural proteins are often essential for viral replication and assembly, making them less suitable targets for rational vaccine marker design. In contrast, LSDV001 represents a conserved virion-associated protein that is not essential for viral replication based on current functional studies and has demonstrated immunogenicity but has been less explored as a diagnostic antigen. The use of such non-classical antigens may expand the range of detectable antibody responses and provide complementary diagnostic value. Accordingly, the LSDV001-based cELISA developed in this study is not intended to replace existing assays, but rather to complement current serological approaches.

Despite these strengths, certain limitations should be acknowledged. The validation panel was primarily composed of serum samples from two geographic regions in China, and further evaluation using sera from broader epidemiological settings will be necessary to confirm the general applicability of the assay (41). In addition, the lack of well-characterized sera positive for sheeppox virus (SPPV) and goatpox virus (GTPV) limited the assessment of potential cross-reactivity within the Capripoxvirus genus. Future studies should include expanded validation and epitope mapping of mAb 8E5 to further define antigenic specificity (23).

In conclusion, the LSDV001-based cELISA provides a practical tool for LSDV serological detection. By expanding antigen selection beyond classical structural proteins, this assay offers a complementary approach for improving serological surveillance and evaluation of vaccination responses. Further optimization and large-scale validation will support its application in diverse field settings.

## Data Availability

The original contributions presented in the study are included in the article/Supplementary material, further inquiries can be directed to the corresponding authors.
