# Supplementary material for: Development of a monoclonal antibody-based competitive ELISA for serological detection of lumpy skin disease virus
Source: Front Vet Sci. 2026 May 20;13:1819650. doi: 10.3389/fvets.2026.1819650 (PMC13229619; doi:10.3389/fvets.2026.1819650)
Supplement: Supplementary file 1 [file Table_1.docx]

Supplementary Materials

Supplementary Table S1. Optimization of coating LSDV001 antigen concentration and serum dilution, with the mAb 8E5 concentration fixed at 1 μg/mL. Data are presented as percentage inhibition (PI) values.

| LSDV001 concentration  (μg/mL) | Serum dilution | | | | |
| --- | --- | --- | --- | --- | --- |
|  | 1:1 | 1:2 | 1:4 | 1:8 | 1:16 |
| 0.625 | 56.3% | 66.2% | 44.4% | 39.4% | 31.2% |
| 1.25 | 66.6% | **70.7%** | 58.8% | 50.9% | 44.6% |
| 2.5 | 50.9% | 51.8% | 40.7% | 38.9% | 35.6% |
| 5 | 43.1% | 44.2% | 42.5% | 39.4% | 30.9% |
| 10 | 39.2% | 41.7% | 37.5% | 33.4% | 30.1% |

Supplementary Table S2. Optimization of mAb 8E5 dilution. Data are presented as percentage inhibition (PI) values.

| PI | mAb 8E5 dilution | | | | | | | | |
| --- | --- | --- | --- | --- | --- | --- | --- | --- | --- |
|  | 1:200 | 1:400 | 1:800 | 1:1600 | 1:3200 | 1:6400 | 1:12800 | 1:25600 |  |
|  | 25.8% | 44.2% | 46.8% | 62.8% | 57.7% | **70.0%** | 65.3% | 59.7% |  |

Supplementary Table S3. Optimaization of blocking Buffer. Data are presented as percentage inhibition (PI) values.

| PI | Optimal Blocking Buffer | | | | | | | |
| --- | --- | --- | --- | --- | --- | --- | --- | --- |
|  | 1% skimmed milk | 3% skimmed milk | 5% skimmed milk | 10% skimmed milk | 1% BSA | Plate stabilizer I | Plate stabilizer II |  |
|  | 54.4% | 58.3% | **66.7%** | 65.9% | 50.1% | 59.2% | 62.9% |  |

Plate stabilizer I and Plate stabilizer II are commercially available blocking reagents.

Supplementary Table S4. Optimization of blocking time. Data are presented as percentage inhibition (PI) values.

| PI | Blocking time | | | |
| --- | --- | --- | --- | --- |
|  | 30 min | 60 min | 90 min | 120 min |
|  | 55.4% | **68.5%** | 64.6% | 58.9% |

Supplementary Table S5. Optimization of incubation time of serum and mAb 8E5. Data are presented as percentage inhibition (PI) values.

| PI | Incubation time | | | |
| --- | --- | --- | --- | --- |
|  | 30 min | 60 min | 90 min | 120 min |
|  | 56.5% | **68.2%** | 65.3% | 60.1% |

Supplementary Table S6. Optimization of the dilution of HRP-conjugated secondary antibody. Data are presented as percentage inhibition (PI) values.

| PI | HRP-conjugated secondary antibody dilution | | | |
| --- | --- | --- | --- | --- |
|  | 1:2000 | 1:5000 | 1:8000 | 1:10000 |
|  | 57.0% | **65.7%** | 63.5% | 61.6% |

Supplementary Table S7. Optimization of the incubation time of HRP-conjugated secondary antibody. Data are presented as percentage inhibition (PI) values.

| PI | HRP-conjugated secondary antibody incubation time | | | |
| --- | --- | --- | --- | --- |
|  | 30 min | 60 min | 90 min | 120 min |
|  | 65.5% | **67.2%** | 59.0% | 50.0% |

Supplementary Table S8. Optimization of chromogenic reaction conditions (time and temperature). Data are presented as percentage inhibition (PI) values.

| PI | Chromogenic reaction time | | | |
| --- | --- | --- | --- | --- |
|  | 5 min | 10 min | 15 min | 20 min |
| 37°C | 47.7% | 61.1% | 58.1% | 49.7% |
| RT | 45.9% | 67.6% | 57.8% | 51.1% |

Note: RT, room temperature.
